# Biosensing the Histamine Producing Potential of Bacteria in Tuna

**DOI:** 10.3389/fmicb.2019.01844

**Published:** 2019-08-27

**Authors:** Marcello Trevisani, Matilde Cecchini, Giorgio Fedrizzi, Alessandra Corradini, Rocco Mancusi, Ibtisam E. Tothill

**Affiliations:** ^1^Laboratory of Food Hygiene, Department of Veterinary Medical Sciences, University of Bologna, Bologna, Italy; ^2^Laboratory of Food Chemistry, Istituto Zooprofilattico Sperimentale della Lombardia e dell’Emilia Romagna “Bruno Ubertini”, Brescia, Italy; ^3^Surface Engineering and Precision Institute, Cranfield University, Cranfield, United Kingdom

**Keywords:** histamine-producing bacteria, amperometric biosensor, tuna, histidine decarboxylase activity, *Morganella psychrotolerans*, *Photobacterium phosphoreum*, microbiological criteria

## Abstract

Histamine poisoning is the most common cause of human foodborne illness due to the consumption of fish products. An enzyme-based amperometric biosensor was developed to be used as a screening tool to detect histamine and histamine-producing bacteria (HPB) in tuna. It was developed by immobilizing histidine decarboxylase and horseradish peroxidase on the surface of screen-printed electrodes through a cross-linking procedure employing glutaraldehyde and bovine serum albumin. The signal generated in presence of histamine at the surface of the electrode was measured by chronoamperometry at in presence of a soluble redox mediator. The sensitivity of the electrode was 1.31–1.59 μA/mM, with a linear range from 2 to 20 μg/ml and detection limit of 0.11 μg/ml. In this study fresh tuna filets purchased in supermarkets in different days (*n* = 8) were analyzed to detect HPB. Samples with different concentration of histamine were analyzed with culture-based counting methods, biosensor and HPLC and also a challenge test was made. Recovery of histamine from cultures and tuna samples was also assessed. The presence of *Morganella psychrotolerans*, *Photobacterium phosphoreum*, *P. damselae* and *Hafnia alvei* was detected using culture- and PCR-based methods. At the time of purchase these tuna samples had histamine concentrations from below the limit of detection (LOD) to 60 μg/g. HPLC and biosensor methods provided similar results in the range from zero to 432 μg/g (correlation coefficient, *R*^2^ = 0.990) and the recovery of histamine from cultures and tuna samples was very high (mean bias −12.69 to 1.63%, with root-mean-square error <12%). These results clearly show that fresh tuna is commonly contaminated with strong HPB. The histamine biosensor can be used by the Food Business Operators as a screening tool to detect their presence and to determine whether their process controls are adequate or not.

## Introduction

Histamine poisoning is the most common cause of human foodborne illness due to the consumption of fish products. During the period 2010–2015, 12 EU Member States reported 176 food-borne outbreaks caused by histamine associated with the consumption of fish and fish products. These outbreaks involved 961 human cases and 104 of hospitalizations (European Food Safety Authority [EFSA], 2017).

The intoxication by histamine occurs after the consumption of food containing biogenic amines, particularly histamine, at concentrations higher than 500 ppm (Gonzaga et al., 2009). Histamine production results from the decarboxylation of free histidine present in a wide variety of pelagic fish. Some bacteria that are present in the gut and on the skin have high histidine-decarboxylase activity. These bacteria are likely introduced into the fish flesh in consequence of post-mortem autolytic changes and inappropriate handling during storage or processing. The European Commission Regulation (EC) No 2073, (2005) set criteria defining the acceptability for fish belonging to the families *Scomberidae* (e.g., tuna, albacore, mackerel), *Scombersocidae* (sauries), *Clupeidae* (e.g., herrings and sardines), *Engraulidae* (anchovies), *Coryphaenidae* (mahi-mahi/dorado) and *Pomatomidae* (bluefish), fixing the limits of histamine at 100 mg/kg (m), with a tolerance up to 200 mg/kg (M) in not more than two units sample out of nine to be sampled from the lots inspected. The U.S. Food & Drug Administration have a 50 mg/kg defect action level for histamine in tuna, mahi-mahi and related fish (FDA, 2011). These limits concern fish and fish products placed on the market during their shelf life.

To meet these criteria, manufacturers have to define performance objectives (POs) in operation management and therefore they should be able to estimate the activity of histamine-producing bacteria (HPB) at relevant stages of the production chain. HPB have been classified, according to their histidine-decarboxylase activity, as weak, medium and strong histamine producers (Bjornsdottir et al., 2009). Most active histamine HPB occurring in fish belong to the family *Enterobacteriaceae* (*Morganella*, *Raoultella*, *Erwinia*, *Proteus* and other) (Cantoni, 2008). Among non-*Enterobacteriaceae*, bacteria of the genus *Photobacterium* (i.e., *P. damselae* and *P. phosphoreum*) showed to have a strong histidine decarboxylase activity (HDC) and were implicated in clinical cases of histamine intoxication (Emborg and Dalgaard, 2006; Landete et al., 2008). Since fish products are normally kept at temperatures next to 0°C, the psychrotolerant HPB can have a major role in outbreaks not related to an interruption of the cold chain. These bacteria include *Morganella morganii*-like bacteria (*Morganella psychrotolerans*) and *P. phosphoreum* (Kanki et al., 2004; Emborg et al., 2006; Prester, 2011). The photobacteria are more often detected in fish under vacuum or CO_2_-enriched atmospheres with an extended the shelf-life (Emborg and Dalgaard, 2006).

Analytical methods for histamine testing in fish include HPLC with fluorescent detection (Duflos et al., 2018) or coupled to Mass Spectroscopy (Kaufmann and Maden, 2018). These methods have high accuracy and precision and can be partially automated, but are labor intensive and costly, requiring skilled technicians and expensive instruments. Biosensors can provide a valid alternative for testing fish at the laboratories of the food business operators, which have to qualify suppliers and monitor the fish quality. Amperometric biosensors are relatively inexpensive and analyses can be made using miniaturized, sensitive and portable devices and disposable screen-printed sensors for the electrochemical detection (Hayat and Marty, 2014). The use of bi-enzymatic biosensors combining amino oxidases with peroxidases (i.e., horseradish peroxidase-HRP) present advantages, since hydrogen peroxide, in presence of a suitable oxidized mediator, such as ferrocenium ion, can be reduced on the electrode surface at low operating potential, avoiding undesirable oxidation of electroactive interferents (Tombelli and Mascini, 1998; Niraj and Pandey, 2012). The use of these biosensors for measurement of histamine produced by HPB in standardized culture media can provide a valuable tool for monitoring the quality of fish. By using biosensors less time is required to conduct experiments and do not use solvents for the extraction process.

Extraction of histamine from fish homogenates and culture media with high temperature and high-pressure treatment represent an interesting approach, since histamine solutions can be sterilized by heating with little degradation (Pratter et al., 1985; McDonald et al., 1990) and biosensors do not require labor-intensive clean-up for water soluble small molecules, such as histamine. Sterilization is also useful to inactivate the histidine decarboxylase that can be released by HPB during refrigerated storage and after freeze-thaw sample preparation (Kanki et al., 2007), which would affect the analytical results.

Herein, a simple (second generation) amperometric screen-printed biosensor was used to assist in the detection of histamine-producing bacteria (HPB) and assess their histidine decarboxylase activity in histidine decarboxylase broth (HD broth). Spike and recovery experiments and challenge tests with HPB were carried out under standardized conditions to demonstrate the validity of histamine biosensors as a screening method for testing fish at the laboratories of the food business operators.

## Materials and Methods

### Reagents and Microbiological Media

Diamine Oxidase (DAO) from Porcine Kidney, Peroxidase Type II from Horseradish (HRP), Albumin from Bovine Serum (BSA), Glutaraldehyde (GA) (25% w/v), potassium hexacyanoferrate (II)-Trihydrate (Reagentplus^®^) [K_4_Fe(CN)_6_⋅3H_2_O], Histamine Dihydrochloride (≥99% TLC), sodium phosphate dibasic anhydrous (Na_2_HPO_4_), sodium phosphate monobasic monohydrate (NaH_2_PO_4_⋅H_2_O), Potassium Chloride (KCl), Potassium Hydroxide (KOH), Hydrochloric Acid (HCl), Trichloroacetic Acid (TCA), n-heptane o-phthalaldehyde (OPA), Histidine, Sodium Chloride (NaCl) and 0.0005% pyridoxal HCl were purchased by Sigma-Aldrich (Milan, Italy). All standard solutions used for the biosensor were prepared in phosphate buffer (0.1 M HPO_4_^−2^/H_2_PO_4–_, pH 7.4, with 0.1 M KCl) in deionized water, obtained by reverse osmosis (RO) using the water purification system New Human Power II (Human Corporation, Korea). Pre-formulated microbiological media (Tryptone Soy Broth, TSB; Tryptone Soy Broth, TSA; Violet Red Bile Glucose Agar, VRBGA; thiosulfate-citrate-bile salts-sucrose agar, TCBS; Iron Agar-Lyngby, IA) and peptone were purchased from Thermo Scientific Oxoid, Basingstoke, United Kingdom).

### Apparatus and Electrodes

Amperometric measurements were carried out with a portable BiPotentiostat/Galvanostat μStat 400 from Dropsens (Oviedo, Spain) connected via a Cable Connector (Dropsens, CAST) to Screen-Printed Carbon Electrodes (SPCEs) (Dropsens, DRP-150). The SPCEs include a carbon working electrode (4 mm diameter), a silver pseudo-reference electrode and a carbon counter electrode. The SPCEs were placed in a methacrylate cell (Dropsens, CFLWCL-CONIC) that hold the samples extract on the surface. The supplied DropView 8400 software for Windows was used to control the instrument, plot measurements and perform the analysis of results.

### Assembly of the Enzyme Electrode

Diamine Oxidase and HRP were immobilized onto the surface of the screen-printed carbon electrode (4 mm diameter) using glutaraldehyde and bovine serum albumin (BSA) cross-linking. 10 mg of DAO and 5 mg of HRP were dissolved each in 100 μl of 0.1 M phosphate buffer. 4 mg of BSA were dissolved in 100 μL solution (70 μl of DAO and 30 μl of HRP). Then 15 μl of the DAO-HRP-BSA solution were mixed with 5 μl of GA 2.5% and soon 10 μl of the mixed solution were placed on working electrode and let dry at room temperature; then washed with phosphate buffer to ensure the removal of any unbound enzyme. The modified electrodes were kept at 4°C in phosphate buffer until further use.

### Histamine Biosensor Calibration and Assay Procedure

Samples and standard solutions of histamine were prepared in phosphate buffer (0.1 M, pH 7.4) containing potassium hexacyanoferrate (II) 50 mM (PBS-Med). One hundred μl of this solution was deposited on the surface of the SPCE and the current was measured at −0.025 V after 500 s, when a steady state was reached. Before sample addition, 100 μl of PBS-Med (blank solution) were placed on the electrode and current was measured at −0.025 V vs. screen printed Ag/AgCl reference electrode. The difference in current intensity between the sample and the blank solutions was calculated and correlated with histamine concentration. Experiments were performed in triplicate (*n* = 3). For the histamine calibration curves, the standards consisted of histamine solutions at different concentrations ranging from 2 to 20 μg/ml, which was chosen as the operative range. Samples were diluted using PBS-Med, to have histamine concentrations that fit in the calibration range of the biosensor. The calibration curves have to be calculated every time a new sensor is built, because even small changes in the cross-linking reaction that is needed to immobilize the enzymes may slightly affect the response (Barbosa et al., 2014). The prepared biosensors were stored in PBS at 4°C when not in use and at the beginning of every batch of analyses a low-level sample containing 5 ng/ml of histamine was tested to check the repeatability of the response (calibration check). Results were compensated for the dilution factor. The fresh fish samples were normally diluted 1:10 (1:20–1:50 at 3–7 days of storage), while the range of dilution for enriched HD broth was from 1:200 to 1:1000.

### Fish Samples

The samples were taken from eight lots of fresh yellowfin tuna (*Thunnus albacares*) filets purchased in two supermarkets. The filets were packed on foam polystyrene trays overwrapped with low-density polyethylene film. The samples were transported within 1 h to the laboratory in an insulated chiller box with cool freezer pack. From each fillet a 25-g portion was used for the isolation of background histamine forming bacteria and the remaining part was cut into 5-g pieces and put in polystyrene weighing dishes. These subsamples were covered with a low-density polyethylene film and stored in insulated boxes on ice chips put in a chilling room at 4°C and analyzed for histamine and for detection of histamine-producing bacteria (HPB). Histamine level was determined amperometrically by biosensor in the tuna sub-samples stored on ice in the insulated box for 1 week.

**FIGURE 1:**
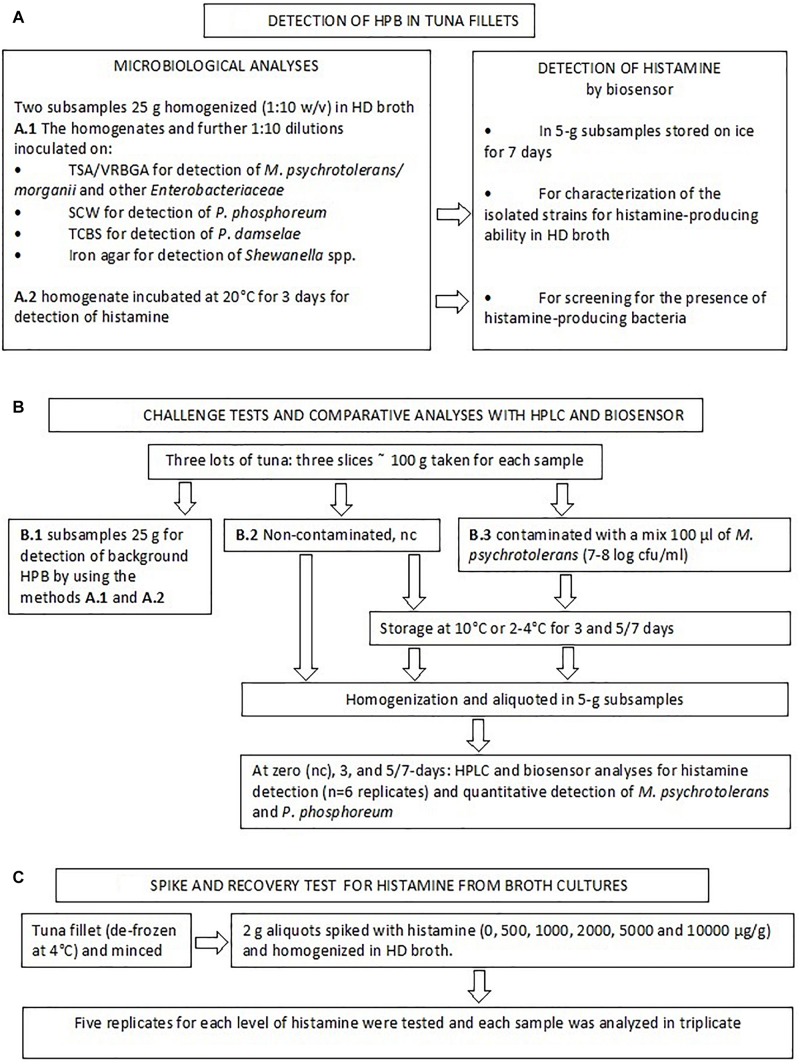
Flow diagram describing the study design.

A flow chart of the analyses that were used to detect and characterize HPB and to validate the biosensor method is reported in Figure 1.

### Methods for Detection of Histamine-Producing Bacteria

Samples were diluted (1:10) in two aliquots of TSB containing 1% histidine, 2% NaCl, and 0.0005% pyridoxal HCl (HD broth). One was incubated at 20°C for 3 days and used for detection of histamine. The other was used for the detection of background histamine-producing bacteria was made by inoculating 1 ml and 0.1 aliquots of the sample homogenates on four different media: (a) TSA/VRBGA; (b) seawater complete medium (SCW medium, composed of peptone 5 g, yeast extract 3 g, glycerol 3 ml, agar 15 g, aged seawater 750 ml, water up to 1 liter) (Martini et al., 2013), (c) TCBS; (d) Iron Agar.

Red colonies on TSA/VRBGA plates were counted and identified as *Morganella psychrotolerans* or *M. morganii* with the method developed by Podeur et al. (2015). Briefly, isolated colonies were enumerated by spreading 1 ml of diluted samples on ≈ 5 ml TSA, then, after 2 h at room temperature, 12–15 ml of VRBGA were added and these TSA/VRBGA plates were incubated (25°C, 2 d). Subsequently, 5 colonies randomly selected from the plates of the highest dilution showing growth and tested for species identification and characterization. SCW plates were incubated at +5°C up to 10 days and plates were also observed in the dark to enumerate luminous *P. phosphoreum* colonies, then five colonies randomly selected were identified using the real-time PCR method developed by Macé et al. (2013). The green colonies that grew on TCBS were tested to detect the presence of *Photobacterium damselae* subsp. *damselae* using the procedure developed by Trevisani et al. (2017). The black colonies that grew on the Iron Agar plates (25°C, 3 days) indicative of H_2_S-producing bacteria were picked and presumptively identified as *Shewanella* spp. by Gram staining and biochemical characteristics (API 20e and API 20NE systems, BioMérieux, France). In addition, non-inoculated samples were analyzed for the presence of HPB by testing the level of histamine produced in HD broth enrichment at 20°C, 2 days). The isolated strains were also evaluated for the presence of histidine decarboxylase gene of the Gram- bacteria (De Las Rivas et al., 2006; Trevisani et al., 2017) and their ability to produce histamine in HD broth. With this aim single colonies isolated from the fish samples (or used for the challenge tests, section “Bacterial Strains Used for the Challenge Tests”) were cultured in duplicate in 10 ml HD broth at 4°C for 5 days (*P. phosphoreum*) or 20°C for 2 days (*M. psychrotolerans* and other HPB) and histamine was extracted as described in the section “Extraction of Histamine From Bacterial Cultures and Recovery Tests” and detected by the enzyme-based amperometric biosensor, as described in the section “Histamine Biosensor Calibration and Assay Procedure.”

### Extraction of Histamine From Bacterial Cultures and Recovery Tests

Histidine decarboxylase broth cultures were sterilized at 121°C for 15 min, then centrifuged (15.000 × *g* for 15 min), diluted in PBS-Med and analyzed with the histamine biosensor.

The extraction efficiency of histamine from broth cultures and tuna samples diluted in phosphate buffer have to be assessed and this was done with spike-recovery tests. With this aim, de-frozen tuna was used to emulate the natural test sample matrix (1:10 tuna in HD broth), but reducing the interference of viable HPB (especially *Photobacterium* spp.) that were often detected in the vacuum-packed fresh tuna samples. Therefore, one fillet of tuna (approximately 100 g) de-frozen at 4°C was minced, 2 g aliquots were spiked with histamine (0, 500, 1000, 2000, 5000, and 10000 μg/g) and homogenized in HD broth. Five replicates for each level of histamine were tested and each sample was analyzed in three replicates. The range 50–1000 μg/ml was decided according to the values of histamine developed in HD broth by low, middle and strong histamine producers (Bjornsdottir et al., 2009). The concentration of histamine was calculated according to the calibration curves constructed for each enzyme electrode. The spike recoveries were calculated by following equation: Spike recovery (%) = (total amount detected−amount original)/amount spiked × 100%.

### Challenge Test With HPB and Comparison of the Biosensor and HPLC Methods for Real Sample Analysis

One aim of this study was to compare the results of the biosensor method with those of an official reference method in a quantification range that is consistent with current legislation. Therefore, in parallel with the above-mentioned analyses a challenge test was made with using the samples of three lots of tuna (Jun 23, Jul 7, and Jul 15) (Figure 1). Three slices of approximately 100 g of tuna were purchased. One was analyzed to detect the presence of background HPB as described at the section Methods for Detection of Histamine-Producing Bacteria.” The other two slices were either inoculated with 100 μl of a *M. psychrotolerans* mixed culture (section “Bacterial Strains Used for the Challenge Tests”) or not inoculated, covered with a low-density polyethylene film and stored at 10°C or 4°C for 3 and 5 or 7 days with the aim of obtaining fish samples with six different levels of histamine in a range between zero and approximately 500 ppm (Table 4). For the analyses the slices were homogenized (aseptically) in a household cutter (Moulinette; Moulinex, Paris, France). The 5-g aliquots were weighted in vessel cleaned with ethanol, rinsed with sterile water and dried in a laminar flow cabinet. The subsamples were analyzed for histamine by the biosensor (*n* = 6) and HPLC (*n* = 6) and for the quantitative detection of *Morganella* spp. and *Photobacterium phosphoreum* (*n* = 2). The 5-g subsamples intended for the analysis with biosensor were diluted (1:10) with phosphate buffer and processed like the bacterial cultures (section “Extraction of Histamine From Bacterial Cultures and Recovery Tests”). The preparation method for HPLC analysis included homogenization of the 5-g subsamples with 60 ml trichloroacetic acid 10% w/v, 2 ml 6N HCl and 4 ml n-heptane, centrifugation at 4000 rpm for 15 min and pre-column derivatization of supernatant with O-phthalaldehyde (OPA). A fluorescence detection system was used; excitation was set at 350 nm and emission was read at 450 nm (EN ISO 19343, 2017). The method also uses 1,7-diaminoheptane internal standard (IS). Quantification of histamine was performed by calculating each response factor against IS and using a calibration curve. LOQ value was 10 μg/g. Detection of other biogenic amines was not considered by the validated method used in this study.

### Bacterial Strains Used for the Challenge Tests

Five strains of *Morganella psychrotolerans*, isolated from different lots of European anchovy (1 strain), European pilchard (2 strains), Atlantic herring (1 strain) and Yellowfin tuna (1 strain) were used (Costanza et al., 2013). All strains were characterized with biochemical tests, are able to grow on ice-cooled fish filets (≈2°C) (Emborg et al., 2006) and were PCR-positive for the gene vasD, specific of the Type VI secretion system of *M. psychrotolerans* (Podeur et al., 2015). In addition, all strains proved to be strong histamine producers in HD broth (approx. 3000 ppm at 20°C in 48 h) (see section “Methods for Detection of Histamine-Producing Bacteria”). The strains were grown in HD broth at 4°C for 4 days and diluted with the same broth to an optical density of approximately 0.39 at 540 nm (≈ 10^8^ CFU/ml). Then they were mixed together and used to inoculate fish samples (1:10 dilution or undiluted).

### Statistical Analysis and Bioanalytical Method Validation

Regression analysis was used to evaluate the linearity of the histamine detection by biosensor in HD broth cultures. The statistical data analysis tools included in Microsoft Excel (Excel 2010, Microsoft, United States) were used for this purpose. The linearity was assessed by visual evaluation of a plot of the difference response ratio versus the respective concentration level. The response function of the calibration curves for histamine in broth cultures (recovery tests) and tuna (challenge tests) was measured by using five calibration standards run in triplicate. The target back-calculated concentrations of the calibration standards have to be within 20% of the nominal value. A calibration check sample spiked at 5 μg/ml was analyzed prior to each batch of analytes being run. Accuracy and repeatability (intra-assay precision) were determined using the spike recovery method. Within-run and between-run accuracy were estimated by analyzing five samples per level at five concentration levels with three runs analyzed in different days. The criteria for acceptability of the data included accuracy and precision within ± 15% deviation (DEV) from the nominal values and precision within 15% relative standard deviation (RSD). The Limit of detection (LOD) was calculated as the concentration corresponding to three times the blank standard deviation and also according to the 3 sb/m criteria, where m is the slope of the linear portion in the calibration graph, and sb was estimated as the standard deviation of the amperometric signals measured (European Medicines Agency [EMA], 2012). Linear regression was used to define the relationship between the concentration of histamine detected by biosensor and HPLC. Regression confidence intervals for the mean concentration were calculated for each sample and the equivalence of results of the two methods was assessed using Bland-Altman diagram for evaluating inter-rating agreement on a continuous scale (Giavarina, 2015) and analysis of the covariance of estimates.

## Results

### Detection of HPB in Tuna Samples

Histamine-producing bacteria were detected in the eight lots of tuna analyzed (Table 1). A high attention was given to test all suspect isolates due to the fact that high histamine concentrations were detected in the enriched samples. *Morganella psychrotolerans* and *Photobacterium phosphoreum* were the HPB most frequently detected, but the second was detected only in the vacuum-packed tuna steaks. Some isolated colonies of *P. phosphoreum* that produced low amount of histamine at 20°C in 2 days, proved to grow better at 4°C. All other strains were able to grow at 4°C, even if their growth rate was not assessed. The presence of the histidine decarboxylase gene (HDC) was detected by PCR in all the strains using the primers designed by De Las Rivas et al. (2006) according to the conserved region from various Gram- HPB, but amplification was very weak with the *P. damselae* strain that produced good amplification levels by using primers that were designed specifically for this species by Trevisani et al. (2017). The *M. psychrotolerans* strains were isolated in two samples from either VRBGA and TCBS.

**TABLE 1:** Histamine-producing bacteria isolated from fresh tuna filets and histamine level after 7 days of storage on ice.

**Lot**	**Histamine detected by biosensor**			**Species**	**PCR**	
	**Culture (μg/ml)^*a*^**	**Tuna 7 days (μg/g)^*b*^**	**Strain (μg/ml)^*c*^**		**id genes^*d*^**	**HDC^*e*^**
Feb 21	5,920	24	8,473^*rt*^	*M. psychrotolerans*	Vas +, Gal k−	+
Mar 7	5,770	18	6,518^*rt*^	*M. psychrotolerans*	Vas +, Gal k−	+
Mar 13	7,880	<LOD	8,506^*rt*^	*P. damselae*		±^∗^
Apr 4	11,046	60	8,212^*rt*^ 368^*rt*^ 7,312^*rt*^	*K. oxytoca H. alvei M. psychrotolerans*	Vas +, Gal k−	+ + +
May 2	1,169	20	88^*rt*^; 332^*ct*^	*P. phosphoreum*	gyrB	+
Jun 23	2,216	<LOD	412^*rt*^; 250^*ct*^	*P. phosphoreum*	gyrB	+
Jul 7	1.451	na	17^*rt*^; 316^*ct*^	*P. phosphoreum*	gyrB	+
Jul 15	na	na	na	*M. psychrotolerans*	Vas +, Gal k−	+

*^*a*^ histamine produced by sample homogenates enriched in HD broth at 20°C, 3 days. ^*b*^ histamine in tuna samples stored at <4°C for 7 days. ^*c*^ histamine produced by isolated colonies enriched in HD broth at 20°C, 2 days (rt) or 4°C, 5 days (ct). ^*d*^ PCR identification test *M. psychrotolerans* (Podeur et al., 2015) and *P. phosphoreum* (Macé et al., 2013). *P. damselae*, *K. oxytoca* and *H. alvei* were identified on the basis of biochemical tests (API 20e and API 20ne). ^*e*^ PCR test for HDC gene of Gram- bacteria (De Las Rivas et al., 2006) or specific of *P. damselae* subsp. *damselae*^∗^ (Trevisani et al., 2017). na, not assessed; <LOD, below the limit of detection.*

### Recovery of Histamine From Broth Cultures

A fundamental challenge in the development of enzyme-modified screen-printed electrodes for assay of histamine is to ensure a high reproducibility from sensor to sensor and the possibility to perform accurate measurements with many samples without significant loss of sensitivity. The results of precision and accuracy of the biosensor-based method are reported in the Table 2. The greatest degree of accuracy was obtained with histamine concentration higher than 5,000 μg/g (500 μg/ml). In the concentration range 497–1991 μg/g (≈50 to 199 μg/ml) the differences between spiked and measured concentrations of histamine were relatively low (mean bias −12.69 to −6.65 percent) but significant.

**TABLE 2:** Precision and accuracy summary table of the method for the quantitative detection of histamine in the enriched broth cultures.

		**Nominal concentration**				
	**N**	**15**	**15**	**15**	**15**	**15**
Spiked^*a*^	Mean (μg/g) ± SD	496.82 ± 1.69	993.17 ± 4.78	1990.67 ± 7.33	4973.68 ± 16.10	9930.49 ± 36.31
Detected^*b*^	Mean (μg/g) ± SD	433.78 ± 16.60	913.50 ± 38.88	1858.28 ± 67.79	4892.79 ± 188.69	10085.79 ± 385.65
Precision	Within-run RSD	9.95%	5.97%	4.85%	7.08%	7.26%
	Between-run RSD	9.93%	4.68%	6.00%	9.98%	9.91%
Accuracy	Mean Bias	−12.69%	−8.02%	−6.65%	−1.62%	1.63%
	RMSE	8.82%	4.40%	5.63%	11.11%	10.20%
	Significance *t*-test	<0.0001	0.00973	0.0116	0.393	0.402

*^*a*^ amount spiked in 2-g tuna samples that were homogenized in HD broth; ^*b*^ values calculated according to the dilution factors.*

Table 3 reports the parameters of calibration curves of the two modified screen-printed electrodes used for the recovery tests. The values of slope and intercept were very close and the coefficients of determinations (*r*-squared) indicated that regression predictions perfectly fit the data. The amperometric response was also verified at every run using check standards (5 μg/ml). The measurements were within 20% of the true value, with RDS of less than 12%. All measurements that were required to assess the recovery from broth cultures (*n* = 225) and for the calibration curves (*n* = 18) were made with only two enzyme electrodes, without loss in sensitivity. The sensitivity of the assembled bi-enzymatic electrodes was good in the range 1.31–1.59 μA/mM (11.81–14.31 nA/μg/ml) with a linear range from 2 to 20 μg/ml and detection limit (LOD) 0.11 μg/ml (Table 3). This LOD value is actually 10×, because samples have to be diluted (1:10). The LOD calculated according to the 3 sb/m criteria for the two electrodes was 1.31 and 1.59 μg/ml.

**TABLE 3:** Calibration curve equations and uncertainty in the regression analysis for the histamine determination in enriched broth cultures of the two m-SPE (A and B) used for the recovery tests.

							**Check standard 5 μg/ml*^*b*^***	
**m-SPE**	**Equation*^*a*^***	***R*^2^**	**S*_*m*_***	**S*_*b*_***	**S_*y,x*_**	**LOD*^*c*^***	**MBE%**	**RMSE%**
A	*y* = −14.31*x* + 6.56	0.996	0.55	3.62	3.46	0.11	−2.83%	11.27%
B	*y* = −11.81*x* + 6.00	1.000	0.30	2.00	1.91	0.11	4.91%	11.83%

**^*a*^* nA vs. concentration (μg/ml); *^*b*^* calibration check standard measured at each run; *^*c*^* calculated as the concentration corresponding to three times the blank standard deviation (μg/ml).*

### Detection of Histamine and HPB in Tuna Samples

Histamine content was assessed in tuna samples stored at <4°C for 7 days (Table 1). The level was always low or below the level of detection, with a maximum of 60 μg/g. One aim of the protocol was to obtain fish samples with six different levels of histamine. Table 4 reports the counts of *M. psychrotolerans* and *P. phosphoreum* in the inoculated and control samples and the levels of histamine measured in these samples using HPLC and the biosensor-based method. Both methods provided similar analytical results in the concentration range 0–432 μg/g. At higher concentrations the biosensor-based method overestimated the concentration of histamine in the samples. It should be noted that heat-induced coagulation of the soluble proteins produced a gel in the soluble phase that must be discarded before diluting the supernatant in PBS-Med to avoid interferences (data not-reported). The biosensor that was used for this validation test did not showed significant loss of sensitivity after 1 month. The sensitivity, 0.653 μA/mM, was good. Its stability was evaluated periodically during this time by measuring the response toward histamine check standard (5 μg/ml) at each run in four different days over a 74-days period demonstrating excellent stability, since the signal decreased by only 10.8% (from 25.34 to 22.61 μA, RSD = 7.89%). The most common problem that required to renew the enzyme electrode was the detachment of the BSA-glutaraldehyde membrane that occurred after extended repeated use, with the enzyme electrodes stored at 4°C and immersed in phosphate buffer solution when not in use.

**TABLE 4:** Levels of histamine (μg/g) and numbers of *M. psychrotolerans* and *P. phosphoreum* (mean ± SD) detected in inoculated and non-inoculated tuna samples at different days of storage.

**Inoculum log CFU/g**	**Storage°C, days**	**Lot**	**Histamine (μg/g)**		**Count (log CFU/g)**	
			**HPLC**	**Biosensor**	***M. psychrotolerans***	***P. phosphoreum***
Non-inoculated	<4°C, 3 days	Jun 23	<LOD	<LOD	ND	NL-NC
	10°C, 3 days	Jul 15	66 ± 5	63 ± 2	3.99 ± 0.10	4.74 ± 0.18
	<4°C, 7 days	Jul 15	107 ± 23	119 ± 15	2.00 ± 0.08	ND
	10°C, 5 days	Jul 7	116 ± 6	95 ± 4	ND	5.05 ± 0.12
3.4 ± 2.6	10°C, 3 days	Jun 23	138 ± 2	138 ± 9	5.72 ± 0.21	5.10 ± 0.19
3.4 ± 2.6	10°C, 7 days	Jun 23	432 ± 46	395 ± 49	7.72 ± 0.02	TNTC
4.4 ± 3.6	10°C, 7 days	Jun 23	517 ± 32^*a*^	753 ± 52^*b*^	7.69 ± 0.08	TNTC

*Histamine values with different superscript in the same raw are significantly different; TNTC, too numerous to count at the dilution 10^–3^, confluent growth tested positive for *gyrB* gene of *P. phosphoreum* by PCR. NL-NC, non-luminescent colonies too numerous to count at the dilution 10^–3^; ND, not detected at the dilution 10^–1^.*

**FIGURE 2:**
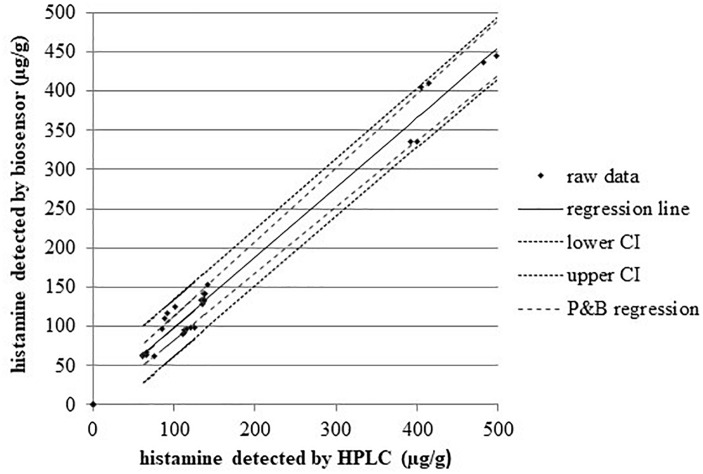
Regression analysis of the two methods for histamine. *n* = 30, concentration range 75–498 μg/g; Pearson correlation coefficient *r* = 0.99, *P* < 0.001. Kendall Tau coefficient 0.85, *P* < 0.001. *R*^2^ = 0.98. Regression line equation: *y* = 8.85 + 0.89*x*; confidence interval CI95% for the intercept –1.70 to 19.41 and for slope 0.84 to 0.94; Passing and Bablok regression (P and B), regression lines have slopes and intercept equal to CI95%.

**FIGURE 3:**
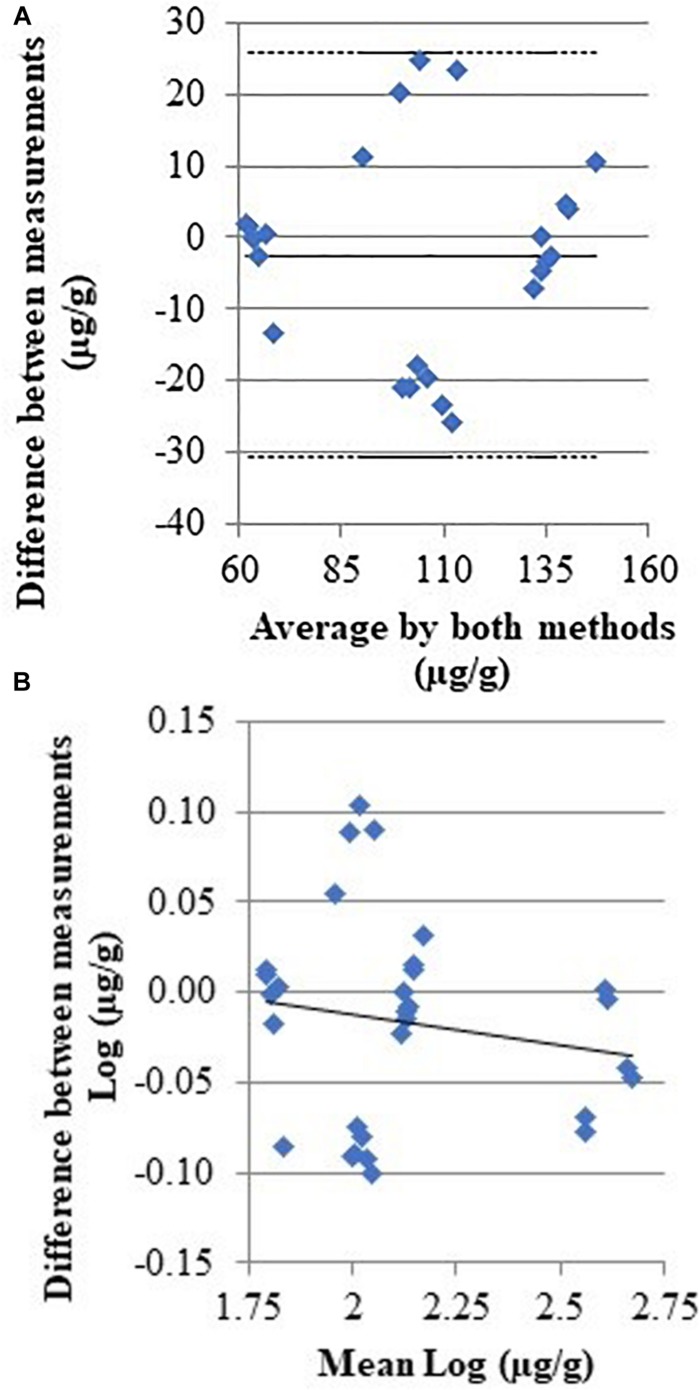
Scatter diagrams with the average and the difference of histamine concentrations detected by HPLC and biosensor. **(A)** Full line indicate the regression line; **(B)** full and dashed lines indicate the mean difference and 95%CI, respectively [CI95% –30.72; 25.74].

Figure 2 displays a scatterplot of the histamine levels measured with both methods in the concentration range 75–498 μg/g (*n* = 6 replicates). The correlation coefficient between the two methods is *r* = 0.990 (95% confidence interval, CI = 0.980–0.995, *P* < 0.001), and the regression equation is *y* = 8.85 (−1.70 to 19.41) + 0.89 (0.84 to 0.94) *x*; that could be evaluated as a very good agreement, with the slope coefficient indicating a relative low underestimation by biosensor. The inter-rating agreement and the Bland-Altman diagram (difference plot) between HPLC and biosensor assays are displayed in the Figure 3.

Histamine was detected in either the inoculated and non-inoculated samples. The natural background microflora of the samples, other than the inoculated *M. psychrotolerans* strains, contributed to produce histamine. The microbiological analyses revealed the presence of *P. phosphoreum* (luminescent strains) in the lots Jul 7 and Jul 15 and *M. psychrotolerans* in the lot Jul 15. Their histamine-producing potential was demonstrated in the HD broth cultures, where the concentration of histamine in the non-inoculated samples of lots Jun 23, Jul 7, and Jul 15 was 2216, 1451, and 849 μg/ml, respectively, after incubation at 10°C for 3 days.

## Discussion

### The Analytical Device

The results of a screening test that revealed the presence of strong HPB in HD broth and this was undoubtedly a good reason to look carefully to their presence using specific culture methods. The enzymatic biosensor was also useful to assess their histidine decarboxylase activity. The presence of high concentration of histamine in HD broth inoculated with tuna samples unequivocally indicated that they were contaminated with strong HPB and or that their number was high. The enzymatic biosensor presents some advantages over other methods that are used for screening, such as the conductimetric method developed by Klausen and Huss (1987). The conductimetric method is specific for some microbial species and requires a microbiological impedance analyzer (Bjornsdottir et al., 2009) that is more expensive than a portable amperometer connected to a personal computer. The histamine biosensor has a superior versatility because it can be used to measure also the histamine content in tuna samples as described in this study. All this data provides information to the Food Business Operators that have to decide on the shelf life, best management practices and have to evaluate the suppliers. The amperometric biosensor used in this study is a “second generation electrochemical biosensor.” Third generation biosensors involve immobilized mediators, acting as non-diffusion redox relay stations, effectively facilitating the transport of electrons from the enzyme active site to the electrode (Putzbach and Ronkainen, 2013). These sensors are required especially for *in vivo* measurements, because mediators do not escape the active layer immobilized on the electrodes and has been developed also for histamine detection, providing faster and higher current response and response (Pérez et al., 2013; Apetrei and Apetrei, 2016). The mediator-free sensor developed by Pérez et al. (2013) for example required 3 min to reach a stable steady state current, while it was 500 s (≈ 8 min) in this study. The sensitivity of the mediator-free sensor also superior (1.31–1.53 vs. 19 μA/mM), but both methods are fit for the purpose, since the range of measurements required for histamine detection in fresh tuna is between ≈ 10 (LOD of the reference method) and 100 mg/kg. It should be noted also that small changes in the sensitivity of biosensors can be observed as a consequence of the BSA-glutaraldehyde cross-linking (in this study) or other enzyme and mediator immobilization methods, thus calibration curves have to be calculated for every biosensor (i.e., for accurate measurements) unless the biosensor production methods are highly standardized. The principal interfering compounds (i.e., substances that produce noticeable amperometric response) that can affect measurements using DAO-HRP based electrodes in tuna samples include other biogenic amines that can be produced as a result of microbial contamination and inadequate storage conditions (Biji et al., 2016). Recently a commercial enzymatic biosensor for histamine detection in fish and fishery products (Biofish-300, Biolan, Zamudio, Spain) obtained AOAC certification (Salleres et al., 2016).

Even if enzymatic methods are specific, other biogenic amines, such as cadaverine and tyramine and especially putrescine, can interfere with histamine biosensors that are based on the diamine oxidase activity (Lange and Wittmann, 2002). Putrescine and cadaverine are produced through decarboxylation of free ornithine and lysine by the exogenous decarboxylase enzymes released by microorganisms associated with seafood (Biji et al., 2016; Comas-Basté et al., 2019). While all biogenic amines were formed in tuna during storage conditions (Rossi et al., 2002; Visciano et al., 2014) and contribute to histamine intoxication, their accumulation is much more related with spoilage. The differences observed in this study between the results of HPLC and biosensor method concerning the sample stored at 10°C for 7 days (Table 4 and Figure 3) might be due to the presence of other biogenic amines. Currently the only biogenic amine for which the maximum limits have been set in the EU and United States is histamine because of its toxicological effects. The official HPLC method for histamine detection used in this study, therefore, did not consider the use of standards for the quantification of other biogenic amines.

Biogenic amine formation in fish is correlated with the growth of microbial strains with high proteolytic enzyme activity, hence the control of biogenic amine formation is mainly focused on controlling the growth of biogenic amines forming bacteria (Gardini et al., 2016). Freshly caught scombrotoxin forming fish typically contain histamine level less than 2 mg/kg (Food and Agriculture Organization of the United Nations/World Health Organization [FAO/WHO], 2013). In a study of Afsharmanesh et al. (2011) the maximum mean concentration of putrescine, cadaverine and histamine in *Thunnus albacares* stored in ice on-board to catch vessels were 23.39, 12.37, and 4.30 μg/g, respectively. A value of 50 μg/g for the sum of histamine, tyramine, putrescine, and cadaverine, which was not exceeded in samples stored at 0°C before organoleptic rejection, was proposed as a guiding limit value for tuna acceptance (Visciano et al., 2012). A value that was higher of this “guiding limit” (60 μg/g) was detected by the histamine biosensor in a tuna sample stored on ice for 7 days, which also had the highest content of histamine detected in enriched HD broth (11,046 μg/g) and was found contaminated by different species of strong HPB, namely *M. psychrotolerans* and *K. oxytoca*).

A histamine biosensor may be also useful to detect also the activity of histidine decarboxylase that has been formed before tuna are frozen, which remains stable in frozen fish and can be reactivated after thawing (Visciano et al., 2014). In de-frozen fish, the number of viable bacteria can be reduced without affecting the activity of the enzyme that has been released into the flesh (Kanki et al., 2007; Economou et al., 2016), thus the measurement of HDC activity in the fish samples might be not always directly correlated with the number of HPB. More recently a histamine amperometric biosensor that is based on the use of antibody-antigen interaction (immunosensor) was developed to provide higher specificity (Dong et al., 2017). Nevertheless, due to the histamine low molecular weight, simple structure and thus low immunogenicity production of high specificity and affinity antibodies is difficult, significant interference with putrescine were reported and only two of the six commercial antibodies tested in a study by Mattsson et al. (2017) bound the histamine free in the solution. Therefore, due to the possible “interferences,” biosensors cannot be used as methods for histamine detection in official controls.

### Detection of HPB and Assessment of Their Activity

With regard to histamine detection in tuna samples, the within-group variance observed in the samples analyzed with the same method was relatively small, but was larger in the samples stored for 5 and 7 days (Table 4). This could be a consequence of differences in the growth rates and decarboxylase activity of HPB that occurred.

All the fresh yellowfin tuna filets used in the comparative tests were found contaminated by either *P. phosphoreum* or *M. psychrotolerans*. The non-inoculated samples of two different lots (Jul 7 and 15) had histamine concentrations equivalent to the legal limit of 100 mg/kg after storage at 10°C for 5 days and 4°C for 7 days, respectively. Notably, the not inoculated aliquots of the lot Jun 23 had undetectable level of histamine after 3 days of storage at 10°C, while its level have reached values >100 mg/kg at the same storage conditions in the aliquot that was inoculated with 3.4 log CFU/g of *M. psychrotolerans*. It was observed also that luminescence of very small colonies of *P. phosphoreum* can be undetectable in the Marine Agar plates. Non-luminescent strains of *P. phosphoreum* has been also reported (Flodgaard et al., 2005). The inoculated samples that were stored at 10°C accumulated histamine reaching concentrations above the legal limit in 3 days and levels higher than 400 mg/kg in 1 week. The number of HPB could be estimated by measurement of their decarboxylase activity in HD broth, but the species that are psychrotolerant, such as *M. psychrotolerans* and *P. phosphoreum*, have different growth rates at the temperatures that are used for the storage of fish (i.e., −1 to 4°C) and at the temperatures that are normally used to measure their histamine-producing ability (i.e., 10 to 25°C) (Morii and Kasama, 2004; Emborg and Dalgaard, 2008; European Food Safety Authority [EFSA], 2015). For example, at 15°C the growth rates of *M. morganii* (mesophilic) and *M. psychrotolerans* are similar and 15°C is also the optimal growth temperature for *P. phosphoreum* (Budsberg et al., 2003). Consequently, specific studies are needed to define the most appropriate temperatures for shelf life evaluation of tuna under appropriate storage temperature conditions. PCR methods that are specific for the most relevant bacteria have been also developed with the aim of detecting quantitatively HPB (Macé et al., 2013; Podeur et al., 2015), but they are also too complex for the scope of the safety management in the fish industries.

## Conclusion

This study showed that the presence of microbial species that are strong histamine-producers is very common in fresh tuna. The ability of enzymatic amperometric biosensor to detect histamine, combined with phosphate buffer extraction under high temperature and pressure, allowed the development of a simple, rapid, and relatively inexpensive method to measure histamine. The histamine biosensor can be used by the Food Business Operators as a screening tool to monitor the microbial contamination in incoming batches of tuna and to make sure that their operations are designed to meet the prescribed Food Safety Objective.

## Data Availability

The raw data supporting the conclusions of this manuscript will be made available by the authors, without undue reservation, to any qualified researcher.

## Author Contributions

MT conceived and designed the study and wrote the manuscript. MC, RM, MT, AC, and GF performed the experiments. All authors discussed the experiment results and reviewed the manuscript.

## Conflict of Interest Statement

The authors declare that the research was conducted in the absence of any commercial or financial relationships that could be construed as a potential conflict of interest. The reviewer GT declared a shared affiliation, with no collaboration, with several of the authors, MT, MC, AC, and RM, to the handling Editor at the time of review.
